# Nanomaterials Driving Technological Advancements in Enhanced Oil Recovery from Low-Permeability Tight Oil Reservoirs: Opportunities and Challenges

**DOI:** 10.3390/nano16080464

**Published:** 2026-04-14

**Authors:** Chengjun Wang, Ge Jin, Weibo Wang, Chao Zhao, Shuo Wang, Yong Zhao, Jun Ni

**Affiliations:** 1 College of Chemistry and Chemical Engineering, Xi’an Shiyou University, Xi’an 710065, China; 2 Shaanxi Key Laboratory of Carbon Dioxide Sequestration and Enhanced Oil Recovery, Xi’an 710075, China; 3 Research Institute of Shaanxi Yanchang Petroleum (Group) Co., Ltd., Xi’an 710075, China; 4 Shaanxi Jingrui Energy Technology Co., Ltd., Xi’an 710018, China

**Keywords:** nanofluid flooding technology, enhanced recovery mechanisms, nanomaterials, nanofluid oil displacement agent, application

## Abstract

Nanofluid flooding technology has demonstrated enormous potential in enhancing the recovery efficiency of unconventional oil and gas resources. However, due to the complex physicochemical properties of nanofluids and their intricate interaction mechanisms in different reservoir environments, the research and application of nanofluids still face numerous challenges. Although existing review articles have systematically covered various aspects of nanofluid flooding technology and its enhanced oil recovery (EOR) mechanisms, they have not comprehensively addressed all facets of nanofluid-based EOR. In particular, they lack detailed introductions to the field applications of nanofluid flooding technology in reservoirs with different geological structural characteristics, the preparation of bio-based nano-oil displacement materials, the technology of forming nanofluids through in situ self-assembly of silica nanoparticles by reservoir microorganisms, and nanomaterial-mediated carbon dioxide flooding and microbial flooding technologies. This paper aims to identify the existing deficiencies in current nanofluid EOR technologies, especially focusing on the green and low-carbon microbial composite nanofluid flooding technology based on the utilization of reservoir microbial resources. Furthermore, targeted future development directions are proposed, with the goal of providing a more comprehensive, in-depth, and forward-looking reference for the theoretical research and industrial application of nanofluid EOR technologies, thereby further promoting the advancement of EOR technologies for low-permeability and tight oil reservoirs.

## 1. Introduction

With the ongoing acceleration of oil and gas exploration and development, domestic reserves of conventional oil and gas resources have declined significantly [1], leading to growing pressure on resource replacement. For decades, China’s oil and gas production has primarily depended on medium- and high-permeability reservoirs, which offer advantages such as high natural productivity, low development costs, and mature technological support-factors that have historically driven the rapid expansion of the industry [2,3]. However, after extensive large-scale exploitation over many years, high-quality reservoirs have been progressively depleted, and newly discovered reserves are increasingly characterized by poorer quality. There is a marked upward trend in the proportion of low-permeability, ultra-low-permeability, and even extremely low-permeability reservoirs among newly proven reserves. According to available data [4], low-permeability oil and gas resources currently account for more than 70% of total national resources and are widely distributed across major hydrocarbon-bearing basins, including the Ordos, Songliao, Junggar, and Sichuan Basins. In particular, tight oil and tight gas have emerged as key contributors to reserve growth and production increases in the unconventional oil and gas sector [5,6].

Against this backdrop, the emphasis in conventional oil and gas exploration and development has progressively shifted from medium- and high-permeability reservoirs to low-permeability reservoirs [7]. This transition reflects not only the objective consequences of changing resource endowment but also the strategic realignment of national energy policy. Low-permeability reservoirs are typically characterized by low porosity, poor permeability, high fluid flow resistance, and extremely limited natural productivity, rendering conventional development methods ineffective for economically viable exploitation [8,9]. As such, their successful development relies on a suite of advanced technologies, including hydraulic fracturing, horizontal drilling, precision waterflooding, and intelligent reservoir management [10,11,12]. In recent years, advancements in shale gas development techniques and the expansion of digital oilfield initiatives have facilitated the adaptation of key technologies to low-permeability reservoirs, leading to significant improvements in both development efficiency and recovery rates [13]. Therefore, achieving economic, efficient, and sustainable development of low-permeability oil and gas reservoirs has become one of the key measures to ensure national energy security [6].

Currently, traditional enhanced oil recovery (EOR) technologies, such as thermal flooding, chemical flooding, and miscible flooding, have been widely applied on a large scale in many oilfields around the world over the past few decades [14,15,16,17,18,19]. After a century of development, the near-surface conventional oil resources are gradually depleting, which has led to a continuous shift in the focus of oil and gas exploration and development towards deep and ultra-deep unconventional reservoirs to meet the growing energy demand. However, the applicability of existing EOR technologies in unconventional reservoirs is significantly limited. The fundamental constraints lie in the typical geological characteristics of these reservoirs, such as high temperature, strong heterogeneity, low porosity, and extremely low permeability. These physical properties directly result in insufficient sweep volume, low microscopic oil displacement efficiency, difficulty in effectively controlling fluid migration paths, and rapid degradation or failure of injected chemicals in high-temperature and high-mineralization environments [15,16]. Therefore, developing a new EOR technology system that is suitable for the characteristics of unconventional reservoirs has become a key issue that the current petroleum industry urgently needs to break through.

**Figure 1:**
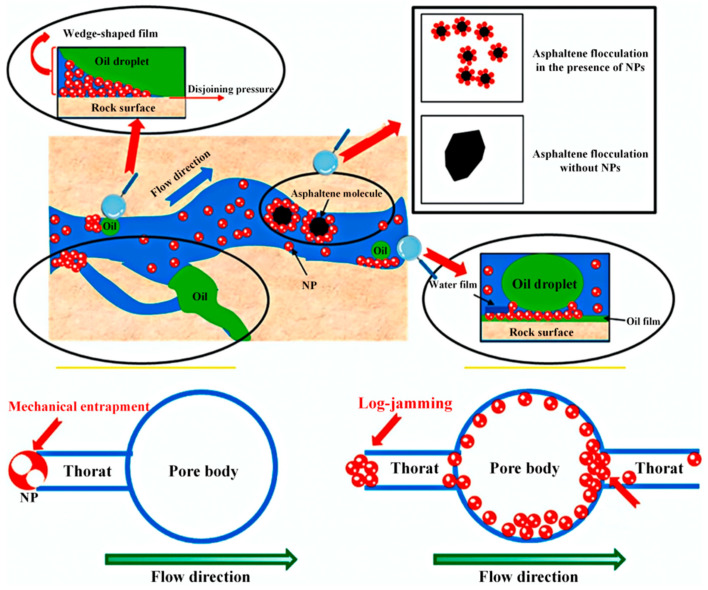
A summary of the primary mechanisms involved in nanotechnology-driven enhanced oil recovery [15].

Among the various technologies employed for EOR in oil reservoirs, nanomaterials have demonstrated considerable application potential owing to their distinctive physical and chemical properties [20]. Nanofluids are stable colloidal dispersions formed by uniformly dispersing nanoparticles with a particle size ranging from 1 to 100 nm in a base carrier fluid (such as water, brine, polymer solution, etc.) and possess multiple technical advantages: (i) the nanoscale endows them with a high specific surface area and strong interfacial activity; (ii) the raw materials are widely available, and the preparation process is mature, offering good cost-effectiveness; (iii) most candidate materials (such as SiO_2_, TiO_2_, and bentonite derivatives) have good environmental compatibility and low ecotoxicity; and (iv) robust structural integrity and colloidal stability are retained under special reservoir conditions (≤120 °C, ≥100,000 mg/L TDS), underscoring outstanding thermal stability and salt tolerance [21]. Owing to their nanoscale dimensions, these particles are capable of entering and migrating through both micrometer- and nanometer-scale pore-throat networks within the reservoir matrix. During the displacement process, nanoparticles adsorb preferentially at the oil–water interface or the rock–fluid interface, effectively reducing interfacial tension (IFT) and altering wettability toward a more water-wet, EOR-favorable condition [22]. The reduction in IFT mitigates capillary forces that trap crude oil in the porous medium, facilitating the mobilization of residual oil; concurrently, the shift toward water-wet wettability improves the imbibition and sweep efficiency of the injected fluid [23]. These synergistic mechanisms collectively enhance fluid flow dynamics in the porous medium (Figure 1), thereby significantly improving crude oil recovery rates [24].

The development of nano-enhanced oil recovery technology has gone through three main stages [25,26]: the early stage of applying nano-surfactants, the stage of nano-composite microemulsion flooding, and the stage of comprehensive application of nanomaterials. Since 2000, this technology has gradually become a research hotspot, marking the beginning of a new era of nano-oil recovery. Currently, the research focus of nanomaterials in oil and gas development mainly lies in nanofluid oil displacement agents and nano-drilling fluids, followed by nanoscale anti-swelling agents for formations, nanotracker, nano-supporting agents, etc. In terms of material types, nano-silica, nano-titanium dioxide, nano-cellulose, polymer nano-microspheres, and nano-graphene are the most widely used nanomaterial categories at present. With the deep integration of materials science, reservoir engineering, and nanotechnology, nano-enhanced oil recovery technology is gradually building a relatively complete theoretical system and technology chain, providing important technical support for the efficient development of low-permeability tight oil reservoirs.

In recent years, scholars at home and abroad have conducted extensive review studies on the application of nanofluids in EOR (Table 1). Davoudi et al. [27] systematically explored the role of different types of nanoparticles in EOR, reviewed the application of nanotechnology in laboratories and fields, and analyzed its economic and environmental impacts. Silka et al. [28] summarized the oil displacement characteristics of various nanoparticles and discussed the prospects of green nanomaterials in EOR, emphasizing their significant advantages in environmental protection and low cost. They also explored the application of nanoparticles in assisting other oil displacement methods. Chakraborty and Panigrahi [29] emphasized the key role of nanofluid stability in EOR and discussed nanofluid stabilization techniques. Lashari et al. [30] reviewed the preparation and stabilization methods of nanofluids, the factors affecting their stability, the mechanism of the impact of stability on IFT and wettability, and the relationship between rheological behavior and nanofluid stability. They also expounded on the current challenges and future research directions in nanofluid stability research. Hou et al. [31] systematically summarized the multiple mechanisms of nanofluids in EOR, particularly highlighting the significant effects of different nanoparticles in improving wettability, and discussed the adsorption of nanoparticles in reservoirs. Tong et al. [32] focused their review on the current research status of the mechanisms of nanofluids in EOR, further emphasizing the importance of research on the surface modification of nanoparticles. Alnarabiji and Husein [33] mainly discussed the application and potential advantages of nanofluids based on bare nanoparticles in EOR. Regarding EOR, they analyzed the performance of these nanoparticles in different environments, including their stability, rheological properties, and action mechanisms. Yakasai et al. [34] emphasized the advantages of nanofluids in EOR, explored the key factors affecting recovery rate, and summarized the main factors influencing the oil displacement efficiency of nanofluids.

Although existing review literature has systematically covered various aspects of the application of nanofluid flooding technology, the stability of nanofluids, and the mechanism of EOR, it has not comprehensively covered all aspects of nanofluid EOR, especially lacking introductions to the application of nanofluid flooding technology in oil reservoirs with different geological structural characteristics, CO_2_ flooding, microbial flooding, and field applications. In addition, the discussion on the EOR mechanism of nanofluid flooding technology is still incomplete. Based on this, this paper aims to provide a comprehensive and up-to-date review of nanofluid flooding technology, examining the widely used nanoparticles or new nanofluid flooding materials in EOR in recent years, and focusing on reviewing the key impacts of different nanofluid flooding materials on the EOR process, as well as discussing the EOR mechanisms mediated by them. Additionally, this paper also lists and discusses the application of nanofluid flooding technology in domestic and foreign oilfields. Finally, the challenges and future research directions of nanofluid EOR are also discussed. This paper will provide theoretical support for the laboratory research and field application of nanofluids and aims to help researchers further promote the development of nanofluid EOR technology.

## 2. EOR Mechanism of Nanomaterials for Oil Displacement Technology

The oil displacement mechanism of nanofluids has significant reservoir adaptability (Table 2), and there are obvious differences in the potential for enhancing recovery rates in different types of oil reservoirs [43]. For a long time, the academic community has conducted systematic research on the core oil displacement mechanisms of nanofluids, such as wettability regulation, interfacial tension reduction, and adsorption and migration of nanoparticles, and has carried out multi-scale experimental verification and numerical simulation evaluation of their EOR effects. In recent years, with the in-depth application of nanofluid oil displacement technology and its synergistic coupling with traditional EOR technologies such as polymer flooding, surfactant flooding, and gas flooding, several new composite oil displacement mechanisms have emerged, such as the synergistic effect of nanoparticles and surfactants and the stabilization of gas foam by nanofluids [44]. Against this background, in-depth analysis of the action paths, key control factors, and applicable boundary conditions of both single and composite oil displacement mechanisms of nanofluids not only helps to improve the theoretical system of nanoscale EOR but also provides scientific basis and technical support for the design of customized oil displacement schemes for complex reservoirs.

### 2.1. Nanoscale-Size Effect and Permeability Enhancement Ability

Nanoparticles exhibit pronounced small-size effects due to their nanoscale dimensions, resulting in physical properties that are fundamentally distinct from those of bulk materials [45]. Furthermore, the small-size effect dramatically increases the specific surface area and the fraction of surface atoms, thereby enhancing surface reactivity, chemical activity, and catalytic efficiency [46]. Collectively, these effects not only profoundly influence dielectric properties, electromagnetic responses, and acousto-optic performance but also fundamentally modify the thermodynamic stability and reaction kinetics of nanomaterials. This provides a critical physical foundation for the rational design and functional optimization of advanced nanomaterials for diverse technological applications.

**Figure 2:**
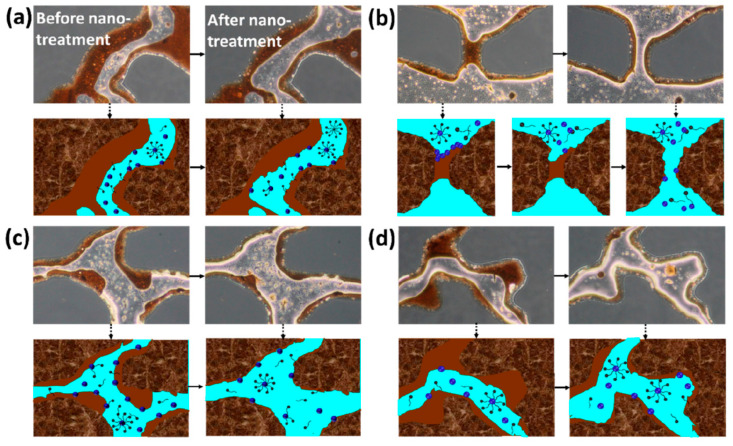
Mechanisms of nano-flooding for four typical residual oil distributions: (**a**) the residual oil on the rock surface, (**b**) the residual oil in the pore throat, (**c**) the residual oil in the connection position of the pore space, (**d**) the residual oil in “dead-end” pores [50].

However, the most significant advantage of nano-scale oil displacement agents lies in their nano-scale size (typically ranging from 1 to 100 nm), which enables them to effectively penetrate into the micro-nano pore-throat systems that conventional oil displacement agents have difficulty reaching [47]. Research indicates that when the particle size is less than one-third of the pore throat diameter, the risk of retention in the reservoir can be significantly reduced, and irreversible blockage can be avoided [48]. The nano-emulsion developed by Jiangsu Oilfield has a molecular diameter of approximately one-thousandth of a human hair (about 100 nm), and it has excellent deep penetration capabilities, allowing it to enter micro-pores with diameters less than 4 microns in ultra-low permeability oil reservoirs [49]. This outstanding seepage characteristic enables nanoparticles to act on residual oil droplets in the microscopic pore structure (Figure 2), thereby significantly enhancing the oil displacement efficiency [50].

During the deep migration process, nanoparticles achieve stepwise profile control through a dynamic behavior of “bridging-temporary plugging-redispersion” [20,50]. When nanoparticles migrate to smaller pore throats, they can form a temporary bridging structure at the throat entrance, causing local plugging and forcing subsequent injected fluids to flow into the uninvaded areas, thereby expanding the macroscopic swept volume [51]. As the injection pressure increases, the bridging structure breaks due to shear force or changes in pore structure, and the nanoparticles re-disperse and continue to migrate deeper into the reservoir [52]. This intelligent flow regulation mechanism endows nano-aided oil displacement agents with the ability to adapt to reservoir heterogeneity, achieving deep fluid flow redirection and uniform advancement. Research has confirmed that nanoparticles can increase the swept volume of low-permeability reservoirs by more than 15%, effectively entering the “blind spots” and dead oil zones that are difficult to reach by conventional water flooding, significantly improving the development effect [50,53,54]. Gazem et al. [55] compared the oil displacement effects of polymer and surfactant solutions containing 0.5% (mass fraction) SiO_2_ nanoparticles and those without nanoparticles. When the segment containing SiO_2_ nanoparticles was injected, both the pressure drop and the recovery rate were higher than those of the pure polymer-surfactant segment. The pressure drop increased by approximately 15 pounds per square inch, and the recovery rate improved by 6.04%.

Additionally, due to the different pore size distributions, the pressure required to form a blocking effect also varied. Chandio et al. [56] found that at low concentrations, nanoparticles could improve wettability and reduce interfacial tension, which was the main mechanism for enhancing recovery. However, at high concentrations, the blocking effect became the dominant mechanism. Although high concentrations of nanoparticles effectively increased oil recovery through the blocking effect, the overall recovery rate was lower than that achieved by multiple synergistic mechanisms at low concentrations. This is because as the concentration of the nanofluid increases, the blocking effect intensifies, but excessive concentration leads to the aggregation of nanoparticles into larger clusters. When these clusters exceed the pore size, the blocking effect gradually transitions to mechanical blockage, resulting in permanent pore blockage. Therefore, since the size of nanoparticles directly affects mechanical blockage and the wicking effect, a detailed study of the pore structure distribution in the reservoir is necessary in applications for enhanced oil recovery. Optimized nanoparticle size and injection strategies can enhance the wicking effect while preventing excessive aggregation, thereby avoiding the transition from the wicking effect to mechanical blockage.

### 2.2. Wettability Reversal Capacity

In the unexploited reservoirs of low-permeability tight oil reservoirs, the rock surfaces have undergone complex physicochemical processes during long-term contact with crude oil, gradually forming oil-wet or strongly oil-wet interfaces. The wettability of reservoir rocks is one of the key factors affecting the efficiency of water flooding development [57]. Wettability alteration technology changes the wettability state of the rock surface, converting the originally oil-wet surface, which is unfavorable for water flooding, into water-wet or neutral wettability, thereby reducing capillary resistance in the pores, enhancing the mobility of the oil phase, and achieving pressure reduction and increased injection, ultimately improving the recovery rate of crude oil [58]. This process is typically characterized by a transition from oil-wet to water-wet or neutral wettability. Due to prolonged interaction with underground crude oil, most reservoir rocks exhibit oil-wet characteristics; on such oil-attracting surfaces, water flooding requires overcoming significant adhesion work and capillary forces, leading to low displacement efficiency.

Additionally, because water has a lower viscosity than oil, injected water tends to flow along the center of the pore channels, leaving oil films on the pore walls, causing fingering and channeling phenomena, which further limit the sweep efficiency and EOR [59]. Research has shown that amphiphilic nanomaterials can regulate wettability through the directional adsorption of their molecular structures: their oil-attracting ends preferentially anchor to the crude oil phase or oil-contaminated surfaces, while the water-attracting ends are exposed towards the water phase, reconstructing the wettability environment of the rock interface and significantly enhancing the mobility of the oil phase [60,61]. Luo et al. [62] revealed the “film-lifting” mechanism of Janus graphene nanosheets in their study—that is, the nanosheets self-assemble at the oil–water interface and push the oil film upwards, breaking the original wettability balance and successfully inducing a transition from oil-wet to water-wet on the rock surface, significantly improving the oil displacement efficiency. Shirazi et al. [63] studied the wettability regulation ability of TiO_2_ nanofluids, and the experimental results showed that this nanofluid could reduce the contact angle of oil-wet cores from 139° to 80°, achieving a transition from moderately strong oil-wet to neutral wettability. Li et al. [64] prepared amphiphilic nano-black card materials by introducing oil-attracting groups to transition metal sulfides for surface modification, which could reduce the contact angle of oil-wet quartz plates from 116° to 91.2°, approaching a neutral wettability state; the mechanism may lie in the entanglement of the oil-attracting groups on the surface of the nanomaterials with oil molecules, promoting the exposure of the water-attracting substrate and altering the interfacial energy distribution. Jafarbeigi et al. [64] synthesized coated and modified halloysite nanotubes (HNTs)/SiO_2_ composite materials using the sol-gel method, and at a mass fraction of only 0.05%, it could significantly reduce the contact angle of oil-wet glass plates from 145.8° to 57.2°, reversing to a water-wet state; this effect is attributed to the increased density of hydroxyl groups on the surface of the modified material, enhancing the interfacial hydrophilicity. In addition, Ngouana et al. [65] investigated the influence of salinity on the improvement of nanoparticle wettability. Within the range of 0 to 20,000 ppm, the degree of wettability improvement increased with the increase in salinity. However, at higher salinity levels, the improvement trend declined due to the suppression of the double electric layer, thereby weakening the electrostatic repulsion of nanoparticles. The mechanism by which nanoparticles alter the wettability of rock surfaces remains a subject of ongoing debate. The specific processes by which different nanoparticles affect the degree of wettability alteration are not yet fully understood. Further experimental research is urgently needed to clarify the underlying principles.

### 2.3. Interface Regulation and Mobility Control Capability

The interfacial tension between oil and water originates from the interfacial contraction driving force in the coexisting two-phase system and is a key physical parameter that affects the efficiency of oilfield development and the effect of enhanced production [66]. Traditionally, reducing interfacial tension mainly relies on adding surfactants to water, but this method is prone to surfactant adsorption on rock surfaces, resulting in chemical agent loss, resource waste, and increased operation costs. Nanoparticles can regulate the intermolecular forces at the interface through their interaction with oil and water molecules. By introducing nanoparticle-based surfactants, the interfacial tension between oil and water can be significantly reduced, weakening the retention ability of the oil phase in the porous medium, thereby promoting the detachment of crude oil from micro-nano pores and its effective displacement [67].

Nanofluid flooding agents not only enhance the utilization efficiency of the agents but also exhibit superior oil displacement performance compared to conventional systems. The mechanism by which they reduce interfacial tension is mainly based on the following two theoretical models [68,69]: (1) Molecular interaction mechanism: When the interface reaches a balanced state, nanofluid flooding agents can self-assemble at the oil–water interface to form a stable composite interfacial film, which is composed of a layered structure of nanomaterials, oil phase and water phase. This significantly reduces the polarity difference between oil and water and enhances the molecular-level interaction between the two phases at the interface. (2) Thermodynamic stability mechanism: Nanofluid flooding agents reduce the interfacial free energy, compensating for the entropy loss caused by the increase in interface area, thereby effectively reducing the interfacial tension. This process helps to weaken capillary resistance, making it easier for the displacing fluid to deform and flow through the pore throats of the formation, thereby improving the microscopic oil displacement efficiency and increasing the crude oil recovery rate. In addition, in the nanofluid-crude oil system, the IFT usually decreases with the increase in the nanofluid concentration, but there is a lower limit to this decrease. Ivanova et al. [70] observed that when the nanofluid concentration was below 0.2 wt%, the IFT could be further reduced on the basis of the reduction by surfactants. However, when the concentration exceeded 0.2 wt%, the IFT would increase. This increase was attributed to the aggregation of nanoparticles, which hindered their further adsorption at the oil–water interface and thus limited the reduction of IFT.

Although the ability of nanoparticles to reduce the interfacial tension between oil and water has been confirmed, the capacity of unmodified nanoparticles to lower the interfacial tension is very limited and far from sufficient to achieve effective crude oil recovery. Therefore, surface modification of nanoparticles has been explored. An effective approach is to graft hydrophilic and hydrophobic groups on the opposite sides of nanoparticles, thereby forming Janus nanoparticles. The hydrophilic end of the modified nanoparticles faces the aqueous phase, while the hydrophobic end faces the oil phase. This arrangement enables the nanoparticles to penetrate the oil–water interface, transforming the fluid-fluid contact into particle-fluid contact, thereby reducing the interfacial tension. Tang et al. [71] compared modified Janus SiO_2_ nanoparticles with unmodified SiO_2_ nanoparticles and found that 0.05% SiO_2_ nanoparticles only reduced the interfacial tension from 69.8 mN/m to 28.4 mN/m, while 0.05% Janus nanoparticles reduced the interfacial tension from 36.4 mN/m to 0.067 mN/m. The reduction in oil–water interfacial tension by nanoparticles is achieved by transforming the two-phase fluid interface into a particle–fluid interface. Therefore, enhancing the adsorption capacity of the fluid interface for nanoparticles is crucial for achieving ultra-low interfacial tension oil displacement. In the future, it is necessary to investigate how the surface modification and electrostatic properties of nanoparticles can improve the stability of fluid interface adsorption under the influence of multiple factors in the pore space.

**Table 2:** Types of nanoparticle materials and their primary mechanisms for EOR [15,21,43,72].

Mechanism Types	Mechanisms for EOR	Types of Nanoparticle Materials
Macroscopic oil displacement mechanism	Change reservoir wettability	SiO_2_, Al_2_O_3_, TiO_2_, Fe_2_O_4_, and carbon nanotubes
	Reduce the interfacial tension between oil and water	SiO_2_, Al_2_O_3_, TiO_2_, and MgO
	Improve the ratio of oil to water flow	Fe_2_O_4_, carbon nanotubes, and graphene SiO_2_, Al_2_O_3_, CuO, TiO_2_, and ZnO
	Reduce asphaltene precipitation	Nanoparticle polymer microspheres, carbon nanofibers, carbon nanotubes, SiO_2_, TiO_2_, and Fe_2_O_4_
Microscopic oil film displacement mechanism	Separation pressure	SiO_2_
	Density difference	Modified SiO_2_

### 2.4. Structural Disjoining Pressure

Structural separation pressure (SDP) is a component of the additional pressure generated by molecular forces in a thin liquid film when nanoparticles in nanofluids come into contact with oil [73]. Specifically, when nanoparticles in nanofluids come into contact with oil, a wedge-shaped thin liquid film is formed, which spontaneously separates the oil droplet from the rock surface. When the film thickness is sufficient to accommodate multiple layers of particles, the nanoparticles tend to arrange themselves regularly within the film, thereby generating additional pressure within the film, namely SDP. Smaller nanoparticles are more likely to enter the micro-regions of the rock surface in contact with the oil droplet, forming a regular particle layer and thus generating a higher SDP [74,75]. At the top of the wedge-shaped cross-section of the three-phase interface of nanoparticles, oil droplets, and rock, the SDP can reach 50 kPa, which is significantly stronger than the adhesion force of the oil droplet, causing it to detach from the rock surface [76]. SDP also varies with the thickness of the wedge-shaped film, and it oscillates periodically with the increase in distance from the vertex of the wedge-shaped film. The SDP generated by a monolayer of nanoparticles in the film is stronger than that of a bilayer. Lim and Wasan [77] studied how the concentration of nanoparticles, temperature, salinity, and surface hydrophilicity affect the inner and outer contact lines of the wedge-shaped film. Their research results indicated that increasing the volume fraction of nanoparticles, temperature, and surface hydrophilicity would accelerate the movement of the inner contact line, while salinity would reduce the osmotic pressure and weaken the diffusion wetting effect of the wedge-shaped film.

Although it has been confirmed that the SDP of nanofluids can enhance the EOR, to achieve a sufficiently high pressure, the concentration of nanofluids needs to exceed 10 vol%, while EOR experiments typically use concentrations below 1 vol% [74,78]. This implies that in most oil recovery processes, the surface diffusion pressure caused by nanoparticles may not be a key factor. Recent studies have indicated that using nanosheets instead of nanoparticles can reduce the demand for high concentrations. Unlike spherical nanoparticles that require concentrations over 10 vol%, nanosheets only need 0.005 weight%. A field test conducted in the Daqing Oilfield in China evaluated a nanofluid formulated with ODA-MoS_2_ nanosheets at a concentration of 0.005 weight%, and the results showed a significant increase in crude oil production and a decrease in water cut [79]. The successful generation of ultra-low interfacial tension by nanofluids based on nanosheets at low mass concentrations for oil recovery provides practical evidence for the feasibility of nanofluids in EOR applications. However, further research is still needed to determine whether these findings are universally applicable. This includes studying whether ultra-low interfacial tension can still effectively displace oil under neutral or oil-wet conditions and at high capillary pressures.

## 3. Advancements in the Development of Nanomaterial-Based Oil Recovery Systems

With the widespread application of nanomaterials across various industries, researchers worldwide have increasingly integrated nanoparticles into EOR systems [80]. By modulating the physicochemical properties of injection fluids or employing surface functionalization of nanoparticles, oil displacement efficiency can be significantly improved, thereby increasing crude oil recovery [81]. Different nanoparticle types exhibit varying degrees of effectiveness depending on reservoir characteristics, demonstrating distinct potential for improving recovery in diverse reservoir environments. Currently, the primary nanomaterials utilized in oilfield applications include nano-SiO_2_, nano-metal oxides, and functionalized carbon-based materials such as carbon nanotubes and graphene (Table 3) [15,21,43,82,83]. However, the synthesis and deployment costs of these nanomaterials vary considerably. Therefore, selecting appropriate nanomaterials based on reservoir geology and production dynamics, along with optimizing their formulation and compatibility, is critical to maximizing economic returns.

### 3.1. Nano-SiO_2_-Based Oil Displacement Agent

Among various nanomaterials for EOR, nano-SiO_2_ is widely regarded as a highly promising candidate for oilfield applications due to its low cost and environmental compatibility [83]. The surface of SiO_2_ nanoparticles is rich in hydroxyl groups, which facilitates surface functionalization and significantly enhances their dispersion stability in aqueous media. Roustaei et al. [84] reported that the injection of silica nanoparticle fluids increased the recovery rates of light oil and medium oil reservoirs by 25.43% and 14.55%, respectively. Contact angle and interfacial tension tests indicated that under the influence of silica nanoparticles, the contact angles of light oil and medium oil reservoirs decreased from 135° and 130° to 66° and 101°, respectively. Meanwhile, the interfacial tension values dropped from 26.5 mN/m (light oil) and 28.3 mN/m (medium oil) to 1.95 mN/m and 7.3 mN/m, respectively. Hendraningrat et al. [85] demonstrated that in a 3.0 wt% NaCl solution, silica nanoparticles effectively stabilize oil-water emulsions and significantly improve oil recovery in Berea sandstone displacement experiments. Furthermore, these nanoparticles reduce oil–water interfacial tension and alter rock wettability toward a more water-wet state. Shang et al. [86] systematically investigated the oil displacement performance of nano-SiO_2_ fluids in low-permeability rock cores and optimized key injection parameters. Their results indicate that nano-SiO_2_ fluids can effectively lower injection pressure and, under optimal conditions, enhance oil recovery by up to 25.41%.

**Figure 3:**
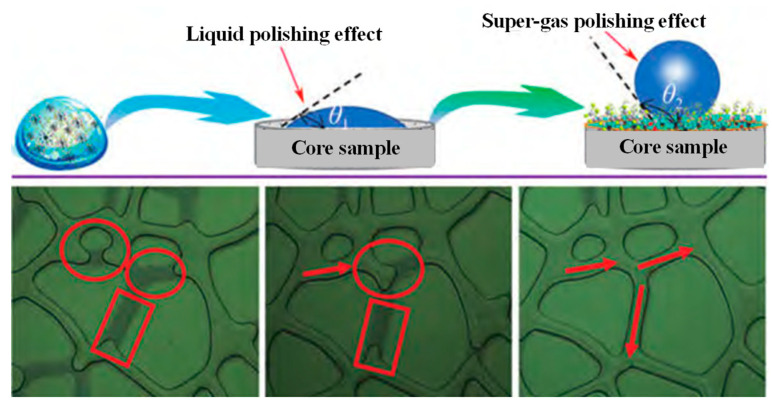
Functionalized nano-SiO_2_ can form a stable wetting adsorption layer [64].

However, due to the intrinsic physical properties of nano-silica, it tends to agglomerate during dispersion, which compromises its application performance. Therefore, surface modification via physical or chemical methods is essential to enhance the colloidal stability of SiO_2_ nanoparticles in aqueous media. Liu et al. [87] systematically reviewed the surface functionalization of nano-SiO_2_ and its role in modulating oil-water-rock three-phase interfacial behavior, using this interfacial regulation as a central theme. In Figure 3, functionalized silica nanoparticles can adsorb onto rock surfaces, effectively altering wettability toward a more water-wet state and thereby promoting crude oil detachment.

Given the inherently weak amphiphilicity of pristine SiO_2_ nanoparticles, recent research has focused on improving their dispersion stability under complex reservoir conditions through precise engineering of surface architecture. In particular, Janus-structured SiO_2_ nanomaterials have emerged as a promising strategy [88]. Shi et al. [89] synthesized asymmetric Janus particles using nano-SiO_2_ as a base material through a Pickering emulsion-assisted approach, followed by fabrication of micro-nano capsules via electrostatic layer-by-layer self-assembly. These Janus micro-nano capsules demonstrated excellent pressure reduction and enhanced injectivity in low-permeability reservoirs. Shang et al. [86] modified hydrophobic nano-SiO_2_ with alkylphenol polyoxyethylene ether, resulting in a nanofluid with high system stability and an oil–water interfacial tension maintained at the 10^−1^ mN/m level. In low-permeability rock cores with a permeability of 20 × 10^−3^ μm^2^, this formulation increased oil recovery by 25.41%. He et al. [90] employed nano-SiO_2_ sol as a carrier and conducted hydrophilic, hydrophobic, and oleophobic surface modifications. The modified materials not only reduced interfacial tension and capillary resistance but also altered the dynamic behavior of water molecules by disrupting hydrogen-bonded network structures, thereby significantly improving water injectivity in ultra-low and super-low permeability reservoirs [91].

Silica-based oil displacement technologies have achieved substantial progress in surface modification strategies, mechanistic understanding, and pilot-scale field applications [92]. To enhance the amphiphilic characteristics of nano-SiO_2_, extensive studies have been conducted globally, including salinization, polymer grafting, metal ion immobilization, and the introduction of polar functional groups (e.g., hydroxyl, carboxyl, and amine groups) [44]. Furthermore, synergistic formulations combining modified SiO_2_ with surfactants are commonly employed: while surfactants effectively reduce oil–water interfacial tension, the emulsions stabilized by amphiphilic silica exhibit superior thermal stability [93]. This dual mechanism enables complementary optimization of interfacial activity and rheological control, leading to enhanced displacement efficiency and improved ultimate oil recovery [60]. Oil recovery experiments have confirmed that the recovery rate of the modified nanoparticles is stable at approximately 86%, while the recovery rates of sodium oleosulfonate and unmodified silica nanoparticles are only 38% and 18% respectively. However, the surface modification of silica nanoparticles may bring environmental uncertainties. Although silica itself is non-toxic, the modification may change its properties, and thus further experimental evaluation is needed.

### 3.2. Metal Oxide Nanoparticle-Based Oil Displacement Agent

#### 3.2.1. Nano-TiO_2_-Based Oil Displacement Agent

TiO_2_ nanomaterials, owing to their low cost, environmental compatibility, and outstanding photocatalytic properties, have gained significant prominence in the field of functional materials [94]. Currently, this material has been widely implemented in engineering applications such as anti-fouling coatings and ultraviolet-shielding composite materials. In recent years, they have gradually been introduced into the research on EOR. In EOR applications, titanium dioxide nanoparticles mainly improve displacement efficiency by reducing interfacial tension and improving rock wettability. With respect to particle morphology, specific surface area, and thermal and pressure stability, TiO_2_ nanomaterials exhibit characteristics comparable to those of SiO_2_ nanomaterials. However, they possess superior surface activity and adsorption capacity, along with a unique capability for photocatalytic degradation of hydrocarbon contaminants. In EOR applications, the mechanisms of TiO_2_ nanomaterials primarily involve two aspects: (1) reducing oil–water interfacial tension to optimize fluid rheological behavior [95] and (2) modifying the wettability of reservoir rocks to facilitate crude oil detachment and displacement [96].

Numerous experimental studies have further validated the efficacy of TiO_2_ nanomaterials in enhancing oil recovery [97]. Ehtesabi et al. [98] conducted experiments to evaluate the ability of titanium dioxide nanofluids in enhancing the recovery of heavy oil in sandstone formations. Their results indicated that even at a concentration as low as 0.01%, titanium dioxide nanofluids could increase the recovery rate from 49% to 80%, strongly demonstrating their significant advantages in improving fluid mobility and oil displacement efficiency. Pu et al. [99] investigated a nanofluid composed of Janus-type titanium dioxide nanoparticles combined with the natural surfactant sucrose stearate. This nanofluid not only significantly improved the wettability of the rock but also increased the oil recovery rate by approximately 10.51% compared with water flooding. Zahedany et al. [100] conducted core flooding experiments and demonstrated that TiO_2_-based nanofluids can elevate ultimate oil recovery to over 80%. Li et al. [101] functionalized TiO_2_ nanomaterials via oleic acid modification and reported a 15% increase in recovery efficiency under low-permeability reservoir conditions when employing an injection formulation containing 0.1% modified nano-TiO_2_ and 0.05% surfactant [102]. Notably, the application of 0.1% carbon nanotube/titanium dioxide nanofluid has been proven to increase oil recovery by more than 10%. These advancements indicate that through composite, hybrid, and synergistic modification strategies, titanium dioxide nanoparticles have great application potential under extreme reservoir conditions, which can reduce the consumption of chemical reagents and achieve more environmentally friendly enhanced oil recovery solutions.

#### 3.2.2. Nano-Fe_3_O_4_-Based Oil Displacement Agent

In recent years, Fe_3_O_4_-nanoparticles have demonstrated significant potential in the field of EOR owing to their unique physicochemical properties [103]. Their high thermal conductivity and superparamagnetic characteristics enable efficient absorption of electromagnetic energy and effective conversion into thermal energy under an external magnetic field [104]. Consequently, upon absorbing electromagnetic radiation, Fe_3_O_4_-nanoparticles can directly generate heat, thereby reducing crude oil viscosity and substantially improving the fluidity of heavy oil [105]. From the standpoint of enhancing crude oil mobility, the synergistic interaction between Fe_3_O_4_-nanoparticles and electromagnetic heating technology offers an innovative approach for optimizing the rheological behavior of crude oil. When an external electromagnetic field is applied, the magnetic nature of Fe_3_O_4_-nanoparticles leads to magnetic hysteresis losses and eddy current losses, resulting in localized heat generation and a consequent rise in reservoir temperature [106]. For instance, under electromagnetic heating conditions, the addition of 0.1 wt% Fe_3_O_4_ nanofluid can reduce the viscosity of crude oil by 20% to 30%, significantly improving the fluidity of high-viscosity crude oil in the reservoir and thereby alleviating the difficulty of its extraction [107]. Paryoto et al. [108] functionalized Fe_3_O_4_ nanoparticle surfaces using citric acid, significantly enhancing nanoparticle dispersion and colloidal stability in crude oil media. Experimental results show that under an applied magnetic field, oil recovery reaches 79.83%, whereas in the absence of a magnetic field, recovery remains at only 35.45%. This marked difference demonstrates that an external magnetic field can drive the directional migration of Fe_3_O_4_-nanoparticles, intensify their interaction with crude oil, further improve fluid transport characteristics, and thereby significantly enhance oil recovery efficiency [109,110]. In the context of foam stabilization for oil displacement, Liu et al. [109] experimentally validated the application potential of Fe_3_O_4_-nanoparticles. They modified Fe_3_O_4_ surfaces using tetraethyl orthosilicate and n-dodecyltrimethoxysilane to tailor surface wettability. By precisely controlling the reagent ratios and reaction conditions, they achieved an optimal balance between hydrophilicity and oleophilicity. The results showed that incorporating the modified Fe_3_O_4_-nanoparticles into the displacement system increased crude oil recovery by approximately 7.1%, offering a novel technical pathway for optimizing EOR processes.

The intrinsic magnetic properties of Fe_3_O_4_-nanoparticles also represent a transformative advancement in oil displacement mechanisms [111]. Their distinct magnetic responsiveness allows for precise spatial manipulation and controlled distribution within porous media under remote magnetic guidance. By modulating the intensity, direction, and duration of the applied magnetic field, the flow trajectory and velocity of magnetic nanofluids can be dynamically regulated, enabling nanoparticles to penetrate regions inaccessible to conventional displacement methods and thereby significantly enhancing reservoir sweep efficiency [108,112]. Divandari et al. [113] investigated the influence of microwave radiation intensity on citric acid-coated Fe_3_O_4_ nanofluids. They found that the oil recovery rate began to change when the magnetic field intensity reached 800 Gauss and increased with the increase in magnetic field intensity, reaching a peak at 2750 Gauss. Additionally, the unique magnetic properties of Fe_3_O_4_ enable remote control of the migration and distribution of nanoparticles within the reservoir, offering new possibilities for enhanced oil recovery. Notably, the application of magnetic fields may pose potential risks to animal behavior and plant growth. Therefore, significant investment is required to ensure the safety of this technology, and prolonged exposure to magnetic fields also incurs substantial energy consumption. Thus, a strict assessment of the environmental benefits and economic feasibility of this technology is imperative.

#### 3.2.3. Nano-Al_2_O_3_-Based Oil Displacement Agent

Al_2_O_3_-nanoparticles, as a key inorganic nanomaterial, typically exist as white powder-like solids with particle sizes ranging from 100 to 200 nm. Owing to their high specific surface area (up to tens or hundreds of m^2^/g), excellent thermal stability (capable of withstanding temperatures above 1000 °C), robust mechanical strength, and chemical inertness, they have been widely utilized in diverse fields such as catalysis, ceramics, and electronics [114]. In recent years, with the advancement of EOR technologies, the application potential of Al_2_O_3_-nanoparticles in this domain has become increasingly evident, leading to a series of significant developments [115].

From the perspective of wettability modulation mechanisms, studies by Zhu et al. [116] demonstrate that Al_2_O_3_-nanoparticles can serve as highly effective wettability modifiers in oil displacement processes. Within reservoir porous media, their pronounced size effect facilitates preferential adsorption at rock–fluid interfaces. Contact angle measurements and wetting/spreading experiments confirm that Al_2_O_3_-based nanofluids significantly alter the wettability of sandstone surfaces. The abundant surface hydroxyl groups form hydrogen bonds with water molecules, enhancing interfacial hydrophilicity and promoting a shift from strongly oil-wet to strongly water-wet conditions. This wettability reversal substantially reduces the adhesion energy between crude oil and the rock surface, thereby facilitating the detachment of trapped oil from pore walls. Experimental results indicate that, compared to conventional chemical flooding agents, the use of Al_2_O_3_ nanofluids increases oil recovery by 15–25%, offering a novel technical approach for improving crude oil mobility.

In the context of high-salinity reservoir development, Akram et al. [117] investigated the synergistic enhancement between Al_2_O_3_-nanoparticles and partially hydrolyzed polyacrylamide under complex formation conditions. Their findings reveal that trace amounts of Al_2_O_3_-nanoparticles can establish physical cross-linking networks with HPAM chains via electrostatic interactions and hydrogen bonding, effectively suppressing polymer chain coiling and aggregation. This interaction significantly enhances the viscosity retention of the polymer solution under saline environments. In a core-scale simulation using a glass bead pack with a permeability of 3.75 μm^2^ as a model porous medium, injection of the Al_2_O_3_/HPAM hybrid system monitored through microscopic visualization technique demonstrated more uniform displacement of residual oil droplets and an expanded sweep volume. The ultimate oil recovery was enhanced by approximately 20–30% relative to the single HPAM system, presenting an innovative strategy for addressing performance degradation in high-salinity reservoirs.

Moreover, the surface chemical properties of Al_2_O_3_-nanoparticles can be precisely tailored through surface modification techniques. Approaches such as silane coupling agent functionalization or polymer grafting enable fine control over surface charge, hydrophilic-lipophilic balance, and colloidal stability, thereby enhancing compatibility with the geological and fluid characteristics of various reservoir types [92]. The integration of material design with optimized EOR processes represents a promising pathway for advancing the large-scale deployment and sustained development of Al_2_O_3_-nanoparticles in enhanced oil recovery applications.

### 3.3. Functionalized Carbon Materials-Based Oil Displacement Agent

Carbon nanomaterials, due to their unique physical and chemical properties, have shown great potential in EOR. They are characterized by small size (<10 nm), availability from a variety of raw materials, good dispersion stability, and resistance to high-temperature and high-salinity environments [118,119,120,121,122]. They particularly exhibit significant advantages under complex reservoir conditions.

#### 3.3.1. Nanofiber-Based Oil Displacement Agent

Cellulose nanofibers are a class of nanoscale polymeric materials derived from natural cellulose through physical, chemical, or biological processes. Owing to their high specific surface area, excellent biocompatibility, low cost, environmental sustainability, and inherent biodegradability, they exhibit significant potential for applications in materials science and energy engineering [123]. Nanocellulose oil displacement agents essentially belong to one-dimensional (1D) nanomaterials. However, in practical applications, they can form two-dimensional (2D) interfacial films through interfacial self-assembly or construct three-dimensional (3D) network structures through intermolecular entanglement and chemical crosslinking, thereby synergistically achieving efficient oil displacement functions. At the oil–water interface, nanocellulose can undergo directional adsorption and ordered self-assembly, forming a dense, high-strength, and highly elastic 2D interfacial film, effectively reducing interfacial tension and inhibiting oil droplet coalescence. Additionally, in the aqueous phase bulk, its large aspect ratio and hydroxyl-rich surface facilitate physical entanglement between fibers and controllable chemical crosslinking, thereby constructing a three-dimensional viscoelastic network with shear-thinning properties, significantly enhancing the apparent viscosity of the system and strengthening the ability to regulate the mobility ratio.

In the field of EOR, Grishkewich et al. [124] have developed a novel nanocellulosic displacement agent based on carboxylated cellulose nanofibers (Carboxylated CNF). The system was constructed by integrating Carboxylated CNF with ethoxylated phytosterols and n-pentanol via intermolecular interactions, resulting in a stable nanostructured fluid system. From a material design perspective, the surface carboxyl groups (–COOH) of Carboxylated CNF can form hydrogen bonds and electrostatic interactions with the polar moieties of ethoxylated phytosterols. Concurrently, n-pentanol acts as a co-surfactant that modulates the interfacial tension and phase behavior of the system. These synergistic effects facilitate the self-assembly of the components into a well-defined microstructure with enhanced colloidal stability [125]. Experimental characterization demonstrates that this displacement system exhibits remarkable salt tolerance under high-salinity conditions. This stability is attributed to the chelation between carboxylate anions and metal cations, which effectively mitigates ion-induced aggregation and preserves the dispersion integrity of the nanofibers [126].

Furthermore, the system spontaneously adsorbs at the oil–water interface, forming a dense and mechanically robust interfacial film that promotes the formation of finely dispersed microemulsions. As a result, the oil–water interfacial tension is significantly reduced from an initial value of 0.5 mN/m to below 0.1 mN/m, thereby lowering the flow resistance of crude oil [127,128]. Core-flooding experiments conducted in low-permeability reservoir models revealed that injecting 0.5 pore volume of the nanofluid enabled effective penetration into micron-scale pore throats [129]. Through a comprehensive mechanism including the reduction of interfacial tension and the alteration of wetting properties, this agent effectively moved and displaced the trapped oil, resulting in an increase of 13.47% in the final recovery rate [130]. These findings not only highlight the promising application potential of nanocellulosic agents in low-permeability reservoirs but also provide valuable theoretical insights and technical guidance for the development of green and high-performance EOR materials. With continued advances in understanding the structure–property relationships of cellulose nanofibers, further optimization of surface functionalization and multi-component formulation strategies may enhance their performance, paving the way for more sustainable and environmentally benign approaches in enhanced oil recovery technologies.

#### 3.3.2. Carbon Nanotubes-Based Oil Displacement Agent

Carbon nanotubes (CNTs) are hollow tubular structures formed by curling single or multiple layers of carbon atoms. They possess a nanometer-scale diameter (high permeability), an extremely high aspect ratio (interface adhesion ability), and excellent mechanical properties (rigid peelability). These characteristics make them suitable for penetrating micro-nano pores in oil and gas reservoirs. By altering the wetting properties of rock surfaces, removing oil films at the peeling boundary, stabilizing the oil–water interface, or regulating fluid flow rates, they can achieve the functions of initiating and displacing microscopic residual oil [131]. In the field of petroleum engineering, CNTs have demonstrated considerable potential for application in EOR due to their unique atomic-scale structure and tunable surface chemistry [132]. Particularly in the development of high-water-cut and low-permeability reservoirs, CNT-based composite displacement systems exhibit synergistic enhancement mechanisms [133]. On one hand, the high specific surface area of CNTs enhances the adsorption capacity of surfactants at the oil–water interface, enabling interfacial tension to be reduced to ultra-low levels (<0.01 mN/m), thereby significantly reducing crude oil flow resistance [134]. On the other hand, owing to their nanoscale dimensions, CNTs can effectively penetrate sub-micron pore throats. Through surface charge modulation and wettability alteration, they promote the transition of rock surfaces from oil-wet to water-wet conditions, enhancing capillary imbibition and facilitating efficient displacement of residual oil [135]. For example, Chen et al. [136] found that the addition of multi-walled carbon nanotubes could increase the oil recovery rate by approximately 4.6% compared with the use of surfactants alone. Alnarabiji et al. [137] tested the oil recovery performance of hydrophobic multi-walled carbon nanotubes at concentrations of 0.01%, 0.05%, and 0.1%, achieving recovery rates of 29.2%, 31.8%, and 23%, respectively. Surprisingly, the higher the concentration, the lower the recovery rate. Furthermore, Afzalitabar et al. [138] conducted a systematic comparison of the EOR performance of hydrophobic multi-walled CNTs, single-walled CNTs, and activated carbon nanoparticles using contact angle measurements and interfacial tensiometry. Their results confirmed that carbon nanotubes significantly modify rock surface wettability, inducing contact angle shifts exceeding 30°, while simultaneously reducing oil–water interfacial tension by more than 50%, thereby markedly improving crude oil mobility and recovery efficiency. With ongoing advances in materials science, further breakthroughs in CNT-enabled EOR are anticipated through rational structural design and optimized multi-component formulations, paving the way for innovation and technological progress in petroleum engineering.

#### 3.3.3. Graphene Oxide-Based Oil Displacement Agent

Graphene oxide (GO), as a representative two-dimensional nanomaterial, features a unique single-atom-thick lattice structure and serves as a functional additive in oil–water separation membranes. It enables the construction of composite membrane systems with high permeability and selective adsorption capacity, thereby enhancing fluid displacement efficiency in oil reservoirs and facilitating efficient oil–water separation. Li et al. [139] systematically investigated a binary EOR system composed of modified graphene oxide and polymers, employing characterization techniques such as interfacial tensiometry and optical microscopy to examine its interfacial behavior, emulsification performance, and displacement efficiency. Experimental results demonstrated that even at an extremely low concentration of 10 mg/L, GO reduced the oil–water interfacial tension to the order of 10^−3^ mN/m. This significant reduction is primarily attributed to the synergistic adsorption between oxygen-containing functional groups on the GO surface and surfactant molecules, along with the ordered self-assembly of two-dimensional nanosheets at the oil–water interface. Flooding experiments further confirmed that the binary composite system increased crude oil recovery by 26.25% compared to conventional methods. The enhancement mechanism involves multiple synergistic effects, including substantial interfacial tension reduction, improved oil emulsification and transport, and wettability alteration of rock surfaces from hydrophobic to hydrophilic.

**Table 3:** Summary of recent research on the application of carbon nanomaterials in enhancing oil recovery [15,43,50,140].

Types	Nanomaterials	Average Particle Size (nm)	Morphology of Nanomaterials	Advantages of EOR
Nano-SiO_2_	SiO_2_	10–15	Sphere	Small particle size, large specific surface area, and high hydrophilicity
Metal oxide nanoparticles	Al_2_O_3_	40	—	High surface activity, increasing the oil flow rate ratio, and reducing the viscosity of crude oil
	TiO_2_	50–70	Sphere	Surface area large and the wettability of the surface changes from oil-wet to water-wet
	Fe_3_O_4_/Fe_2_O_3_	20–40	Approximately spherical	Superparamagnetic, small particle size and easily separable
Nanoparticle polymer microspheres	Separation pressure	40–500	Sphere	Strong viscoelasticity, good thermal stability, excellent salt resistance, and good swelling performance
Carbon nanomaterials	Carbon nanofibers	—	Rod-shaped fibers	High shear resistance
	Carbon nanotubes	—	Tubular fibers	Strong corrosion resistance and suitable for high-temperature and high-pressure oil reservoirs
	Graphene oxide	—	Sheet-like fibers	Reduces the interfacial tension between oil and water and alters the wettability of the reservoir

Moreover, Li et al. [141] developed a graphene oxide nanomaterial-reinforced polymer flooding system and conducted comprehensive evaluations of its rheological properties and long-term stability through rheological analysis, shear resistance testing, and accelerated aging experiments. The results revealed that, compared to conventional polymer systems, the composite formulation containing 0.15 wt% GO exhibited superior viscosity enhancement, enhanced shear resistance, and improved thermal–chemical stability. Under simulated reservoir conditions, the system effectively improved the mobility control capability of the polymer solution, expanded the sweep volume, and ultimately increased oil recovery by 5.04% [142]. These findings not only establish a robust theoretical foundation and technical basis for the application of GO-based materials in petroleum engineering but also highlight their promising potential in advancing green and high-efficiency EOR technologies. With continued advances in understanding material–fluid interactions, future developments through structural engineering and multiphase composite design are expected to yield intelligent, high-performance flooding systems, driving the evolution of EOR toward greater precision, efficiency, and sustainability.

### 3.4. Bio-Based Nano Oil Displacement Agent

Bio-based nanoscale oil displacement materials represent an important frontier direction in the field of EOR in recent years [140]. Through rational design, these materials combine natural bio-based components (such as cellulose nanocrystals, chitosan, sodium alginate, etc.) with functional inorganic nanounits (such as silica, titanium dioxide, magnetic ferrite, etc.) through structural composites or interface coupling. While retaining inherent advantages such as renewability, biodegradability, and low ecological toxicity, they significantly enhance their dispersion stability, interfacial activity, and rock surface response capabilities in high-temperature, high-salinity, and highly mineralized reservoir environments. This provides a green and sustainable technical path to overcome the bottlenecks of traditional chemical oil displacement agents, such as insufficient temperature and salt resistance, difficult degradation of residues, and potential ecological risks. Table 4 summarizes the recent research on the application of bio-based nanofluid oil displacement agents in EOR.

Bio-based nano-scale oil displacement materials mainly enhance crude oil recovery through the following five types of synergistic mechanisms [50,141]: (1) Wettability control: By adsorbing onto the rock surface and altering its surface free energy, it can reversibly transform the wettability from oil-wet to water-wet or intermediate wettability, thereby weakening the adsorption force of crude oil on the pore wall and promoting the mobilization of residual oil. (2) Significantly reduced interfacial tension: It can form highly efficient interfacial active structures in the oil-water system, reducing the interfacial tension to an ultra-low level (typical values reaching the 10^−3^ mN/m range), effectively weakening capillary resistance and improving the microscopic oil displacement efficiency. (3) Mobility ratio optimization: By leveraging the synergistic thickening and elastic network construction capabilities of nanoparticles with polymers/surfactants, it increases the viscosity and viscoelasticity of the displacing phase, inhibits viscous fingering, and enhances the macroscopic sweep uniformity. (4) Emulsification and viscosity reduction synergy: It has excellent in situ emulsification ability for heavy oil, capable of forming thermodynamically or kinetically stable oil-in-water (O/W) emulsions, with viscosity reduction rates reaching over 90%, significantly improving the flowability of heavy crude oil in porous media. (5) Intelligent temporary plugging and fluid flow diversion: With its controllable particle size distribution and surface functionalization characteristics, it can form reversible and adjustable physical temporary plugging at the throats of high-permeability channels, inducing subsequent injected fluids to divert to low-permeability regions, thereby expanding the macroscopic sweep volume and enhancing the utilization degree.

**Figure 4:**
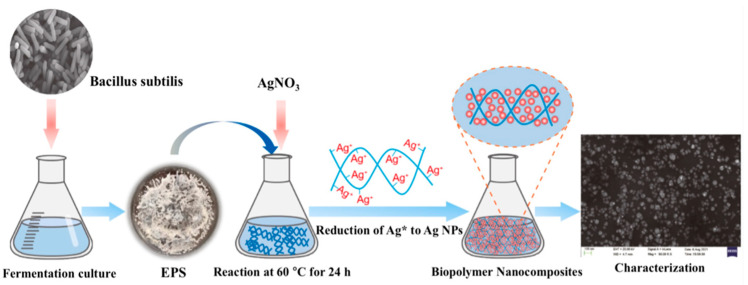
Schematic diagram of the biosynthetic process for the preparation of biopolymer nanocomposite fluids E-Ag NPs. Reproduced from Ref. [143], under the terms of the Creative Commons Attribution 4.0 International License.

Bio-based nano-scale oil displacement materials have gradually moved from the laboratory research stage to the field pilot test stage and have demonstrated good engineering applicability and potential for increasing production [141]. For instance, Wang et al. [142] used the extracellular polysaccharide (EPS) produced by Bacillus subtilis as a reducing agent and stabilizer to synthesize the biopolymer nanocomposite E-Ag NPs in one step (Figure 4). The experimental results showed that the average particle size of Ag NPs synthesized using EPS was between 20 and 50 nanometers. The presence of EPS resulted in a higher Zeta potential value (−43.4 mV), indicating improved stability of the nanofluid and effective inhibition of nanoparticle aggregation. The interfacial tension and wettability evaluation experiments demonstrated that E-Ag NPs performed well in reducing interfacial tension and altering wettability. In the enhanced oil recovery evaluation experiments, the imbibition experiment using E-Ag NPs achieved a maximum recovery rate of 54.32%, and the core displacement experiment reached a maximum recovery rate of 16.33%. Additionally, in a pilot test of nano-cellulose-based water-based dispersion profile control and oil displacement in a low-permeability reservoir block of Changqing Oilfield in China (with an average air permeability of 2.72 × 10^−3^ μm^2^), the comprehensive water cut of the target well group (KS07 well group) significantly decreased from 93.0% to 79.2%, with a cumulative oil increment of 8.0 × 10^3^ m^3^, and the measure’s effective period lasted for 125 days. These cases confirm its huge potential in improving the development effect of medium and low permeability, high-water-cut reservoirs [143].

### 3.5. Reservoir Microbial In Situ Self-Assembled Silica Nanoparticles for Nanofluid Flooding Technology

**Figure 5:**
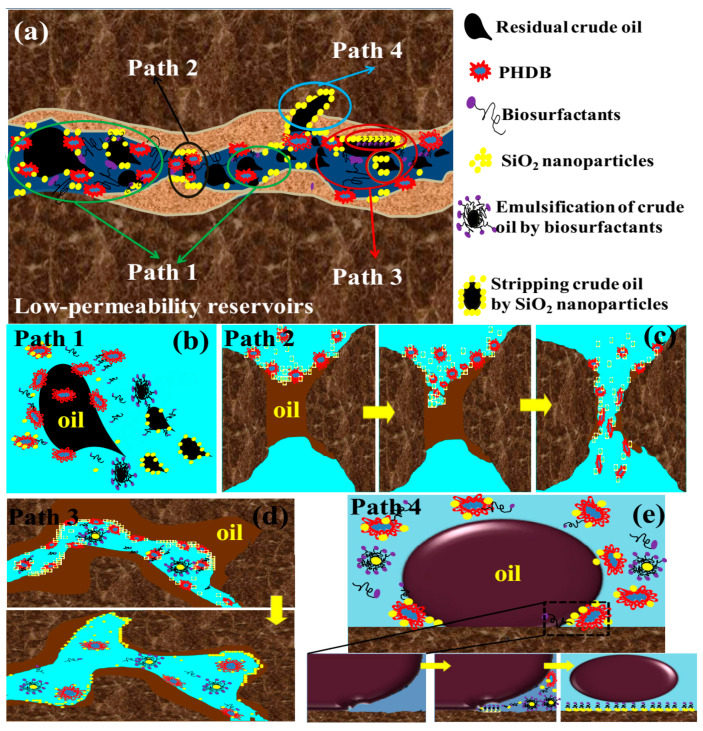
Schematic diagram of the mechanism of PHDB-SiO_2_ nanoparticle composite flooding for enhancing oil recovery. (**a**) Collaborative pathways for EOR by PHDB-SiO_2_ nanoparticle composite displacement. (**b**) Emulsification and dispersion of residual crude oil. (**c**) The clogging channels for the residual oil were dredged by PHDB-SiO_2_ nanoparticle composite displacement. (**d**) The residual crude in the dead-end pores were displaced by PHDB-SiO_2_ nanoparticle composite displacement. (**e**) The residual crude oil being stripped and displaced from the rockface by PHDB-SiO_2_ nanoparticle composite displacement. Reproduced from Ref. [20], under the terms of the Creative Commons Attribution 4.0 International License.

Currently, nanomaterial oil displacement agents and “nano+” composite fluid flooding systems generally face technical challenges in practical applications, such as nanoparticle agglomeration and plugging in near-wellbore zones, as well as effective concentration attenuation during deep migration. Meanwhile, traditional chemical flooding is associated with high carbon emissions, high energy consumption, and prominent environmental risks, whereas single microbial flooding universally suffers from limitations including a long period, limited oil production increase, difficulty in achieving deep migration, and failure to realize synergistic oil increment through profile control and plugging. Based on the utilization of reservoir microbial resources, the technology of forming nanofluids via in situ self-assembly by reservoir microorganisms has emerged as a novel and promising EOR technology. This technology combines the advantages of microbial enhanced oil recovery and nanofluid flooding, utilizing the metabolic activities of indigenous reservoir microorganisms (such as petroleum hydrocarbon-degrading bacteria, PHDB) to achieve in situ synthesis and self-assembly of silica nanoparticles. It not only avoids the problems of agglomeration, sedimentation, and high costs caused by artificially synthesized nanoparticles and surface modification but also exerts the synergistic EOR effect of microorganisms and silica nanoparticles (Figure 5). This paper systematically elaborates on the self-assembly principle, synergistic EOR mechanism, application prospects, and existing deficiencies of this technology, providing a theoretical basis and technical reference for its industrial application in low-permeability and tight oil reservoirs.

(i) Self-assembly principle of silica nanoparticles formed by reservoir microorganisms for nanofluid formation. The in situ self-assembly of silica nanoparticles by reservoir microorganisms is a complex biological-geochemical process, which is closely related to the metabolic activities of microorganisms, the composition of reservoir fluids, and the physical and chemical properties of reservoir rocks. The core principle is that indigenous microorganisms in the reservoir (mainly including *Bacillus cereus*, *Shewanella putrefaciens*, and anaerobic methanotrophic consortia) can secrete specific biomacromolecules (such as extracellular polysaccharides, proteins, and lipids) through their own metabolic activities under suitable reservoir conditions (temperature 40–80 °C, pressure 10–50 MPa, salinity 1000–20,000 ppm), which act as templates and stabilizers to induce the in situ precipitation and self-assembly of dissolved silica in reservoir formation water into stable silica nanoparticles and further form a uniform and stable nanofluid system with reservoir fluids.
(ii) Synergistic oil displacement mechanism of nanofluids formed by reservoir microorganisms and silica nanoparticles. The nanofluid formed by the in situ self-assembly of reservoir microorganisms and silica nanoparticles exerts a synergistic EOR effect through the combined action of microorganisms, silica nanoparticles, and their metabolic products, which is significantly different from the single EOR mechanism of traditional MEOR or nanofluid flooding. The synergistic EOR mechanism mainly includes four aspects: wettability reversal, interface tension reduction, structural disjoining pressure enhancement, and pore throat plugging and profile control, which complement each other and jointly improve the oil recovery efficiency of low-permeability and tight oil reservoirs.
(iii) Application prospects and deficiencies of the technology. The technology of forming nanofluids through in situ self-assembly of silica nanoparticles by reservoir microorganisms is a novel EOR technology that combines the advantages of MEOR and nanofluid flooding. Its unique self-assembly principle realizes the in situ synthesis and stable dispersion of silica nanoparticles, avoiding the defects of traditional artificially synthesized nanoparticles. The synergistic EOR mechanism of microorganisms and silica nanoparticles (wettability reversal, interface tension reduction, structural disjoining pressure enhancement, and pore throat plugging and profile control) can effectively improve the oil recovery efficiency of low-permeability and tight oil reservoirs. The technology has broad application prospects in terms of adaptability, environmental friendliness, and economic feasibility and has important theoretical and practical significance for the efficient development of low-permeability and tight oil resources. However, the technology still has many deficiencies, such as an unclear self-assembly mechanism under complex reservoir conditions, poor controllability of nanofluid properties, insufficient long-term stability research, and unclear environmental impact. In the future, it is necessary to strengthen the research on the self-assembly mechanism of silica nanoparticles by reservoir microorganisms under complex reservoir conditions, develop effective methods to regulate the properties of nanofluids, carry out long-term field tests to evaluate the long-term stability and effectiveness of the technology, and systematically evaluate the environmental impact of the technology, so as to promote the industrial application of the technology and provide new technical support for the efficient and green development of global oil resources.

### 3.6. Application of Different Reservoir Characteristics

Nanofluid-EOR technology has demonstrated significant applicability in different reservoirs by taking advantage of the unique properties of nanoparticles. Table 5 summarizes the research on the application of nanofluid flooding technology in enhancing oil recovery in different types of oil reservoirs. In conventional sandstone reservoirs, it can reduce the oil-water IFT and alter wettability. The nano water-alternating-gas (NWAG) method can enhance oil recovery and achieve carbon dioxide sequestration. For low-permeability and tight reservoirs, it can lower the injection threshold and alleviate water lock. Smart Janus nanosheets and modified nanofluids have achieved good results in practical applications. In carbonate reservoirs, the oil-wet rocks can be transformed into neutral-wet by a mixed system, and the displacement efficiency can be improved. For heavy oil and fractured reservoirs, temperature-salinity responsive nanomaterials can be used to reduce viscosity, block channels, and prevent water breakthrough. The latest frontier research focuses on functionalized nanoparticles, optimized mixed formulations for high-temperature and high-salinity reservoirs (such as surfactant-nanoparticle-polymer systems), and pore-scale mechanism studies to promote large-scale application.

## 4. Efficiency of On-Site Application in Oilfields

### 4.1. Typical Application Case of Oilfields in China

In China, major oilfields such as Changqing, Yanchang, and Bohai have successively conducted research and field trials on nano-displacement technology for EOR [144,145]. Between 2019 and 2020, a pilot-scale “10-injector, 36-producer” field test was implemented in the JiYuan ultra-low permeability reservoir of the Changqing Oilfield, demonstrating favorable performance characterized by increased fluid and oil production, as well as a reduced decline rate [25]. Daqing Oilfield conducted laboratory-scale core flooding experiments using nuclear magnetic resonance techniques to evaluate nano-displacement efficiency in peripheral ultra-low permeability tight reservoirs, with results indicating a significant improvement in recovery factor. In 2019, Yanchang Petroleum carried out a field test of “two injection and one production” with a super-nano strong depressurization displacement agent in the Yongjin 103 and Yongjin 198 well groups, and the corresponding wells increased production by 19.8% [146]. In 2020, a “1-injector, 4-producer” field test of a nano-microsphere displacement agent was conducted in the QSS-46 well group of Wuqi Oilfield. The injection pressure was reduced by 30–40%, and the daily liquid production of the corresponding oil well increased from 5.9 tons to 6.9 tons, with a growth rate of 17%. Meanwhile, the comprehensive water cut dropped from 70.0% to 56.6% [147]. CNOOC has advanced the development and application of nano-displacement technology in offshore oilfields, including Penglai 19-3 Oilfield in the Bohai Sea [148]. Notably, in 2017, a nano-dispersant flooding pilot test was conducted in the conventional heavy oil Q Oilfield, resulting in a 3–10% reduction in water cut and a cumulative incremental oil production of 6158 m^3^ over six months [149].

In addition, the synergistic application of nano-EOR technology and CO_2_ flooding has become an important frontier direction in the current EOR field. In Daqing Oilfield, for low-energy reservoirs in the middle and late stages of development such as the Tai 1 block and the 105 block, the “CO_2_–nano composite flooding” technology research and on-site demonstration project have been deployed, aiming to alleviate the development contradictions such as the continuous decline in formation pressure and the accelerated natural decline. Currently, this technology has entered the stage of large-scale on-site application, with the first phase planning to implement enhanced production services in five wells. Meanwhile, Changqing Oilfield is systematically conducting research on the supercritical CO_2_–nano poly-silicon composite flooding system, mainly targeting tight and low-permeability reservoirs. Laboratory core displacement experiments show that under miscible or near-miscible conditions, this composite system can increase the ultimate recovery rate from the water flooding baseline of 30% to over 50–60%, with an increase of 20–30%, demonstrating a significant synergistic enhanced displacement effect [150].

From the perspective of reservoir types involved in field trials in China, current applications of nano-displacement technology are primarily focused on ultra-low permeability reservoirs, with additional implementation in certain offshore medium-to-high permeability sandstone reservoirs and conventional heavy oilfields. Analysis of oil production enhancement indicates that incremental oil output is closely correlated with reservoir properties: better reservoir quality and higher permeability generally yield greater incremental oil production, whereas ultra-low permeability reservoirs typically exhibit more limited gains [101]. These findings suggest that nano-displacement technology is not only applicable to low- and ultra-low permeability reservoirs but also demonstrates superior performance in medium-to-high permeability systems when deployed under suitable conditions. At present, nano-EOR technology remains predominantly in the stages of laboratory investigation and field piloting. Further reduction in the manufacturing cost of nanomaterials will be critical to enabling large-scale commercial deployment and broader industrial adoption.

### 4.2. Application Case of Oilfields Abroad

Field trials of nanomaterial-based oil displacement technology have been successively conducted in Colombia, Saudi Arabia, Japan, and the United States. In Colombia, the Cupiagua oilfield implemented a pioneering field test targeting asphaltene precipitation inhibition [151]. During the experiment, 220 barrels of alumina nanoparticles equivalent to the amount of oil were injected into the reservoir within eight months, significantly increasing the permeability of the formation and increasing the cumulative oil production by over 300 barrels. Building on this success, the technology was extended to the Castilla and Chichimene heavy oil reservoirs to enhance crude oil mobility and alter formation wettability [152]. Preliminary laboratory studies demonstrated that nanofluids can effectively improve oil recovery through multiple mechanisms, including crude oil viscosity reduction, suppression of asphaltene deposition, and modification of rock wettability. At the Castilla Oilfield, two wells injected with 200 and 150 barrels of nanofluid, respectively, achieved incremental oil production rates of 270 and 280 barrels per day [153]. In the Chichimene Oilfield, following injections of 86 and 107 barrels of nanofluid, the corresponding production increases reached 310 and 87 barrels per day. Monitoring data shows that the viscosity of the crude oil in the two oilfields decreased by 47% on the 9th day and by 70% to 80% on the 30th day after treatment [153]. Variations in crude oil properties between the two reservoirs significantly influenced the duration of nanofluid effectiveness, the extent of viscosity reduction, and overall production enhancement.

In Saudi Arabia, a pilot-scale field test was carried out in the Ghawar Oilfield using an observation well pre-conditioned with 120 barrels of diesel [154]. A total of 255 barrels of carbon-based nanomaterial ADots with a concentration of 130 ppm were injected, and the oil recovery rate increased by 70–80%. Beyond enhanced oil production, the nanoparticles exhibited excellent system stability. Leveraging these findings, a two-year multi-well field study was conducted under extreme reservoir conditions (up to 100 °C, salinity of 150,000 ppm, and pressure of 3200 psi) to assess the long-term performance of ADots in the Ghawar Oilfield [155]. Results confirmed that ADots maintain stable transport and flow characteristics over industrially relevant timeframes.

The Sarucawa Oilfield was the first site in Japan to conduct an EOR field test [156]. This test utilized surface-modified silica nanoparticles, which increased oil recovery through mechanisms such as altering wettability, reducing interfacial tension, generating surface deposits, and creating a blocking effect. During the two-month injection period, approximately 5.5 tons of modified silica nanoparticles with an average concentration of about 0.44% (by weight) were injected. After the injection of the nanofluid, the previously declining oil production curve was effectively stabilized, and oil production significantly increased. A slight increase in the content of silica nanoparticles was detected in the produced fluid, but the majority of the nanoparticles remained in the reservoir. This indicates that some of the injected nanoparticles adhered to the rock surface, improving wettability and ultimately enhancing oil recovery.

In North America’s Bakken tight oil region, functional nanofluid imbibition technology has been trialed for enhanced oil recovery [157]. Temperature- and salt-resistant nanomaterials—such as surface-modified silicon quantum dots—were incorporated into fracturing fluids to promote spontaneous imbibition during shut-in periods, thereby improving matrix oil mobilization. Field data indicate that wells treated with nanofluid-assisted fracturing achieved a 30% higher initial production rate and a 25% lower decline rate compared to conventionally fractured wells [158]. These results strongly validate the technical feasibility and promising application potential of nanomaterials in the development of unconventional tight oil reservoirs.

### 4.3. Potential Defects in On-Site Applications

Nanofluid flooding has been extensively trialed globally for EOR, with promising results but notable limitations that demand critical evaluation. Domestically, major Chinese oilfields (Changqing, Yanchang, Bohai) have conducted pilot tests, mainly focusing on ultra-low permeability reservoirs, with additional applications in offshore medium-to-high permeability and heavy oilfields [144,145,146,147,148,149]. While trials achieved positive outcomes, such as 19.8% production increase in Yanchang, 40% injection pressure reduction in Wuqi, and 6158 m^3^ cumulative oil gain in Bohai [146,147,148,149], synergistic CO_2_-nano-flooding in Daqing and Changqing showed exceptional laboratory recovery gains (up to 30%) [149], which may not fully translate to long-term field performance. The key lies in the fact that the production increase effect is closely related to the quality of the reservoir: the higher the permeability, the more significant the production increase effect will be. However, for ultra-low permeability reservoirs, the production increase effect is very limited [101], indicating that it lacks universal applicability.

Internationally, trials in Colombia, Saudi Arabia, Japan, and the U.S. also present mixed implications. The Kupiagua, Castilla, and Chichimené oilfields in Colombia reported that after the implementation of the technology, the cumulative oil production increased by more than 300 barrels, and the viscosity decreased by up to 50% [151,159]. However, these production increases may be the result of the combined effect of asphalt inhibition and wettability alteration, rather than the sole effect of the nanofluid. The cumulative recovery rate of the Gharwa Oilfield in Saudi Arabia has increased by 70–80% in a short period of time [154]. This is an abnormal short-term effect and does not represent a sustainable improvement in recovery rate technology. Japan’s Sarucawa trial revealed nanoparticle retention, raising pore-blocking risks [156], while U.S. Bakken’s 30% production increase [157] relies on nanofluid-fracturing synergy, not pure nanoflooding. Overall, many high-yield data conflate nanofluid contributions with auxiliary effects. Long-term stability, reservoir-specific adaptability, and high manufacturing costs remain unaddressed globally. Standardized evaluation criteria are urgently needed to validate nanofluid efficacy and facilitate large-scale commercialization.

## 5. Future Challenges and Development Trends in Nanotechnology-EOR

### 5.1. Reservoir Compatibility in Complex Oilfield Systems

As oil reservoir development progresses, reservoir conditions are becoming increasingly complex. High-temperature, high-pressure, and high-mineralization environments pose significant challenges to the stability of nanomaterials, and the long-term stability and adaptability of nanomaterial-based oil displacement technologies in complex reservoirs continue to face substantial technical barriers. Under high-temperature and high-salinity conditions, nanoparticles are prone to agglomeration, sedimentation, and alterations in surface properties, leading to reduced oil displacement efficiency [160]. Therefore, future research should prioritize the investigation of nanoparticle behavior and underlying mechanisms under complex reservoir conditions, with a focus on enhancing overall performance through composite modification with functional materials. Furthermore, significant adsorption and retention of nanoparticles in porous media result in material loss, which not only diminishes utilization efficiency but may also induce micro-pore plugging and other forms of formation damage, particularly in low-permeability reservoirs. Consequently, improving nanoparticle recovery rates and mobility while minimizing formation damage represents a key and urgent research direction for future technological development.

In addition, during the process of promoting the large-scale field application of nano-enhanced oil recovery technology, it is urgently necessary to carry out systematic risk identification and engineering applicability assessment. Its main limitations and potential risks are concentrated in three aspects: First, there is the risk of reservoir damage. During the injection process, nanoparticles may cause irreversible adsorption, agglomeration, and deposition, or even filter cake formation at the pore throats due to their wide particle size distribution, mismatch between the surface zeta potential and formation water, or the bridging effect of divalent cations such as Ca^2+^ and Mg^2+^, leading to a decrease in effective permeability. This risk is particularly significant in low-permeability tight sandstone and carbonate reservoirs with well-developed micro-fractures. Laboratory core flow experiments have shown that in high-salinity environments with a total mineralization degree higher than 10^5^ mg/L and a divalent ion concentration exceeding 500 mg/L, some SiO_2_-based nanofluids can cause 15–30% permeability damage. Lu et al. [161] further confirmed that when the concentration of SiO_2_ nanofluids increased from 0.01 wt% to 0.50 wt%, the retention rate in sandstone cores rose from 7.60% to 80%, corresponding to a permeability loss rate as high as 90%.

Second, there is insufficient environmental stability. Nano-dispersed systems are prone to surface modification layer degradation, colloidal sedimentation, and inactivation of interfacial active components under harsh reservoir conditions such as high temperatures (>90 °C), strong acids/alkalis (pH < 4 or pH > 10), high shear rates (>100 s^−1^), and ultra-high pressures (>70 MPa), thereby weakening their long-term displacement performance. For instance, in a dynamic flow test simulating the conditions of a deep well in the Tarim Basin (bottom hole temperature 128 °C, pressure 78 MPa), unmodified polymer-coated SiO_2_ nanoparticles showed a 42.3% increase in oil–water interfacial tension and a more than 60% decrease in the stability index of O/W emulsions after continuous injection for 48 h, significantly restricting their continuous viscosity reduction and emulsification capabilities.

Third, there are still knowledge gaps. Currently, there is a lack of quantitative characterization and model support for fundamental scientific issues such as the migration path of nanoparticles in porous media, spatial retention distribution characteristics, and the interfacial adsorption-desorption kinetics mechanism with heavy components of crude oil (such as asphaltenes and resins). At the same time, standardized evaluation systems and threshold criteria for environmental safety indicators such as long-term migration potential in underground environments, groundwater exposure risks, ecological availability, and biodegradation behavior have not yet been established. Therefore, future research should focus on the reservoir-adaptive design of nanomaterials (such as temperature-sensitive/salt-sensitive responsive surface functionalization), fine-tuning of injection processes (including slug structure optimization, preflush liquid synergy matching, and injection rate window definition), and the construction of multi-level reservoir damage quantification evaluation methods covering micro-pore scale to macro-well network scale, thereby providing a solid scientific basis and engineering guarantee for the safe, efficient, and sustainable industrial application of this technology.

### 5.2. Environmental Safety

With the widespread application of technologies such as nano-drilling, nano-shaping, and nano-tracing in enhancing crude oil recovery rates, a large amount of functional nanomaterials have been injected into underground reservoirs. While these materials play a role in enhancing efficiency, they may also undergo migration or transformation or remain in the environment under complex geological conditions, posing potential threats to the ecosystem. On one hand, nanoparticles may undergo vertical or lateral migration through the pore structure of the formation, breaking through the aquiclude and entering shallow aquifers, thereby causing groundwater pollution. Particles with a diameter of less than 30 nanometers have been proven to migrate over several hundred meters in sandstone media, presenting a significant environmental diffusion risk. On the other hand, nanoparticles that return to the surface along with the produced fluid constitute another type of environmental challenge. The composition of oilfield-produced water is complex, containing residual crude oil, emulsifiers, polymers, and inorganic salts, as well as the injected nanomaterials. These nanoparticles are prone to adsorption, complexation, or bridging with metal ions, organic macromolecules, or colloidal substances in the aqueous phase, forming stable composite aggregates, which alters their surface properties and sedimentation behavior. This not only affects their stability in the aqueous phase but also significantly increases the difficulty of subsequent treatment.

Given the potential environmental persistence and bioaccumulation of traditional nanomaterials, the development of green, degradable nanomaterials has become an important direction. In recent years, bio-synthesized nanoparticles have attracted widespread attention due to their sustainable preparation pathways and low environmental load [162]. These materials can be generated by reducing metal ions through plant extracts (such as tea, aloe vera, and ginkgo leaves) or multiple types of microorganisms (such as bacteria, fungi, yeasts, etc.), with the preparation process requiring no strong reducing agents or toxic solvents, in line with the principles of green chemistry. The surface of the obtained nanoparticles is usually coated with natural organic layers, giving them good water solubility, colloid stability, and biocompatibility and making them more easily biodegradable or chemically inactivated in the natural environment, significantly reducing the long-term environmental accumulation risk. Studies have shown that iron-based nanoparticles synthesized by Bacillus subtilis can be gradually mineralized by the microbial community within several weeks after completing the oil recovery task and will not persist in the soil or groundwater for a long time. Moreover, degradable nano-carriers constructed based on natural high-molecular substances such as cellulose, chitosan, or starch have been used to develop intelligent responsive oil recovery systems [162], which can release active components after being released under specific temperatures or pH conditions and then automatically decompose, achieving the environmental design concept of “use and then destroy”.

Although nanotechnology provides strong technical support for the efficient development of oil and gas fields, the environmental safety throughout the entire life cycle of nanotechnology urgently requires systematic assessment. Future research should further improve the migration-transformation-attractiveness model of nanomaterials in the underground environment, establish standardized ecological toxicity testing methods, and promote the transformation of green nanomaterials from laboratory research to large-scale engineering applications, in order to achieve coordination and balance between energy efficient development and environmental protection.

### 5.3. Cost Optimization

The synthesis of nano-enhanced oil recovery materials relies on high-precision processes and specialized equipment, with stringent fabrication requirements leading to relatively high production costs [163,164]. Furthermore, due to limitations in current process maturity and scalability, nanomaterials have not yet achieved large-scale, cost-effective commercial production, thereby constraining their widespread deployment in oilfield applications. According to available estimates, the cost of nano-EOR agents may account for 40% to 60% of total project investment, significantly exceeding that of conventional chemical flooding methods [165]. This elevated cost structure poses a critical barrier to the broader adoption of nanomaterials in enhanced oil recovery technologies, particularly in large-scale field implementations, where material expenditure directly impacts economic viability.

Future research should prioritize advancements in process optimization and technological innovation to enhance production efficiency and ensure consistent product quality. Concurrently, efforts should focus on developing more economically viable and environmentally sustainable synthesis pathways to accelerate the engineering-scale application of low-cost, high-performance nano-EOR materials. Moreover, evaluating the feasibility of using naturally occurring or industrially derived nanomaterials (such as bentonite, fly ash and biochar) in enhanced oil recovery not only offers the possibility of significantly reducing raw material costs but also supports the principles of resource recovery and a circular economy. This represents a strategic approach to achieving cost-effectiveness and environmental sustainability [58].

### 5.4. Significance of This Study

Although existing review articles have systematically covered various aspects of nanofluid flooding technology and its EOR mechanisms, they have not comprehensively addressed all facets of nanofluid-based EOR. In particular, they lack detailed introductions to the field applications of nanofluid flooding technology in reservoirs with different geological structural characteristics, the preparation of bio-based nano-oil displacement materials, the technology of forming nanofluids through in situ self-assembly of silica nanoparticles by reservoir microorganisms, and nanomaterial-mediated carbon dioxide flooding and microbial flooding technologies. This paper aims to identify the existing deficiencies in current nanofluid EOR technologies, with a specific focus on the green and low-carbon microbial composite nanofluid flooding technology based on the utilization of reservoir microbial resources. Meanwhile, it systematically summarizes the research and development progress of novel nanomaterials in the past five years (Table 6) [61,166,167,168,169,170], improves the classification system and performance evaluation standards of nano-oil displacement materials, and enriches the application theory of nanomaterials in the EOR field. It not only provides guidance for subsequent basic research and engineering applications but also promotes the development of green and efficient nanofluid flooding technology, offering new ideas and support for the sustainable development of global oil resources.

Firstly, this review addresses the insufficient application of nanofluid flooding technology in reservoirs with complex geological conditions, which is a key bottleneck restricting its industrialization. Most existing studies on nanofluid EOR are carried out under laboratory-simulated conditions with simple geological characteristics, ignoring the complexity and heterogeneity of actual reservoir environments, such as high temperature, high pressure, high salinity, narrow pore throats, and diverse rock mineral compositions. These complex geological factors easily lead to problems such as nanoparticle agglomeration, sedimentation, and effective concentration attenuation during migration, significantly reducing the EOR effect. For example, in high-salinity reservoirs, the compression of the electric double layer will destroy the dispersion stability of nanofluids; in low-permeability tight reservoirs with narrow pore throats, nanoparticles are prone to plugging, causing reservoir damage. However, existing research has not yet formed a systematic solution to adapt nanofluid technology to complex geological conditions, and the application effect of nanofluids in different types of reservoirs with distinct geological characteristics is still unclear. This review focuses on the application dilemma of nanofluid EOR technology in complex geological environments, sorts out the influence rules of different geological factors on nanofluid performance and EOR effect, and provides targeted technical guidance for the adaptation of nanofluid technology to complex reservoirs, which is crucial for breaking the bottleneck of industrial application.

Secondly, this review highlights the novelty of introducing field applications of several emerging nanofluid-related EOR technologies, which is a key innovation compared with existing reviews. Specifically, this review focuses on three novel technologies: the preparation of bio-based nano-oil displacement materials, the technology of forming nanofluids through in situ self-assembly of silica nanoparticles by reservoir microorganisms, and nanomaterial-mediated CO_2_ flooding and microbial flooding technologies. Bio-based nano-oil displacement materials, as green and low-carbon materials, have the advantages of biodegradability, low environmental risk, and low cost, which can solve the environmental pollution problem of traditional chemical nanomaterials, but their field application reports are scattered and not systematically summarized in existing reviews. The technology of in situ self-assembled silica nanoparticles by reservoir microorganisms avoids the agglomeration and high cost problems of artificially synthesized nanoparticles, realizing the in situ synthesis and stable migration of nanofluids, but its field application effect and optimization measures are still lacking systematic combing. Nanomaterial-mediated CO_2_ flooding and microbial flooding technologies combine the advantages of nanofluids with other EOR technologies, significantly improving the EOR effect, but existing reviews have not comprehensively introduced their field application cases and synergistic mechanisms. This review systematically summarizes the field application status, technical advantages, existing problems, and optimization measures of these three technologies, which not only enriches the research content of nanofluid EOR technology but also provides practical reference for their industrial application, highlighting the novelty and practical value of this review.

Finally, to address the bottlenecks of nanofluid flooding technology in industrial application and promote its sustainable development, constructing a “mechanism synergy-material adaptation-reservoir response-cost optimization” four-dimensional analysis framework is proposed as a core strategy. This framework integrates the intrinsic EOR mechanisms, nanomaterial properties, reservoir geological characteristics, and economic feasibility, forming a systematic and integrated analysis system that breaks the isolation of single-factor research and realizes multi-dimensional coordination. The implementation of this framework relies on three key steps: first, conducting multi-mechanism coupling experiments to quantify the synergy degree and establish a mechanism evaluation model; second, building a material-reservoir matching database based on big data, combining machine learning to predict optimal nanomaterial types and parameters; and third, integrating real-time monitoring technologies (logging, core testing) to track reservoir response and developing a cost-benefit evaluation model to realize dynamic optimization of the nanofluid system. This framework provides systematic guidance for the intelligent design and efficient application of nanofluid flooding technology, advancing its industrialization in low-permeability and tight reservoirs.

### 5.5. Prospects

As the development of high-water-cut and high-permeability oil and gas reservoirs in China becomes increasingly challenging, the exploitation of low-permeability and tight oil reservoirs is emerging as a strategic focus in the field of petroleum engineering. Within the framework of tertiary oil recovery, the research and development of novel materials and advanced technologies have become pivotal to enhancing crude oil recovery efficiency. Among these innovations, nano-enabled EOR technologies offer promising new pathways for improving displacement performance. However, under complex geological conditions, the efficient development of low-permeability and tight reservoirs still faces significant technical and economic challenges.

(i) The stability and underlying mechanisms of nanomaterials remain insufficiently understood. Most current studies are conducted under controlled laboratory conditions, whereas actual reservoir environments are far more complex. There remains a critical knowledge gap regarding the migration, distribution, and interfacial behavior of nanomaterials in porous media. Moreover, existing research tends to emphasize single mechanistic effects, with limited systematic investigation into the synergistic interactions among multiple mechanisms. Future efforts should integrate advanced analytical techniques, such as interfacial tension measurements, zeta potential analysis, and in situ microscopic imaging, to systematically characterize the impact of various nanomaterials on oil–water interfacial properties. Coupled with molecular dynamics simulations, these approaches can enable the rational design and microscale analysis of structure–property relationships, thereby deepening the understanding of nanomaterial action mechanisms and facilitating predictive modeling of their subsurface behavior [25].
(ii) Nanomaterials must withstand harsh reservoir conditions. Low-permeability and tight reservoirs often feature extreme environments characterized by elevated temperatures, high salinity, wide pH fluctuations, and strong shear forces. These conditions demand that nanomaterials maintain excellent dispersibility and functional integrity under dynamic operational stresses, avoiding aggregation or deactivation. To enhance environmental adaptability, surface functionalization strategies can be employed, such as introducing steric hindrance groups or ion stabilizers, to improve salt tolerance, thermal stability, and shear resistance. Furthermore, compatibility assessments between nanomaterials and conventional chemical agents (e.g., polymers, surfactants) should be conducted to develop composite systems capable of ensuring long-term stability and sustained performance in reservoir settings.
(iii) High production costs hinder the large-scale deployment of nanomaterials. Current synthesis and modification processes are often intricate and rely on expensive raw materials, limiting industrial scalability. It is therefore imperative to advance key preparation technologies, enhance production efficiency, and ensure consistent product quality to promote the engineering-scale application of low-cost, high-performance nano-EOR agents. Concurrently, alternative strategies based on economic viability and sustainability should be explored, including the evaluation of naturally occurring minerals (e.g., bentonite, kaolin) or industrial by-products (e.g., fly ash, steel slag micropowder) as sources of functional nanocomponents for EOR applications. Such approaches not only reduce material costs but also support resource recycling and circular economy principles, achieving dual objectives of cost reduction and environmental sustainability [58].

In summary, the future trajectory of oilfield displacement technologies is rapidly evolving toward an integrated paradigm of “intelligent responsiveness” and “multifunctional integration.” Owing to their unique physicochemical properties, nanomaterials have become central enablers of this technological transformation [171]. From the perspective of intelligent design, nanomaterials exhibit exceptionally high specific surface areas and abundant surface-active sites, offering inherent advantages for dynamic reservoir management. By taking advantage of the principles in supramolecular chemistry (such as molecular self-assembly, non-covalent interactions, etc.), researchers can precisely design stimulus-responsive nanomaterials with flexible structures. These materials do not possess fixed interfacial packing densities; instead, they dynamically reconfigure in response to real-time reservoir conditions (e.g., pore structure heterogeneity, fluid velocity gradients, oil–water ratio variations). For instance, upon entering high-permeability zones, surface-active components may spontaneously aggregate via supramolecular forces to form dense interfacial barriers, enabling “intelligent plugging” of preferential flow channels. Conversely, in low-permeability regions, conformational relaxation reduces packing density and flow resistance, facilitating uniform displacement front advancement. This “on-demand profile control” mechanism holds transformative potential to overcome the longstanding limitation of limited sweep efficiency in conventional EOR methods, significantly expanding reservoir contact and improving access to residual oil accumulations.

At the multifunctional level, the abundance of modifiable surface functional groups (e.g., hydroxyl, carboxyl, amino) on nanomaterials provides a versatile platform for integrating multiple displacement functions within a single particle. Through precise chemical engineering, a “single-particle, multi-function” architecture can be realized: On one hand, tailored functional groups can establish specific interactions with heavy crude components such as asphaltenes and resins, effectively desorbing oil films from rock surfaces and reducing oil–rock adhesion. On the other hand, nanoparticles can be engineered for targeted capture of dispersed oil droplets—by tuning surface wettability, they act as “microscopic oil scavengers,” adsorbing micron-scale droplets and promoting coalescence through particle-mediated aggregation, ultimately forming mobile “oil trains” or continuous oil banks [172,173]. This end-to-end functional synergy from separation to capture and subsequent fusion not only enhances the efficiency of oil release but can also mobilize isolated oil droplets on a large scale, fundamentally overcoming the inherent limitations of incomplete displacement and droplet retention in traditional methods. This represents a paradigm shift in EOR technology, signaling the transition from passive displacement to active, intelligent, and integrated nanoscale intervention

## 6. Conclusions

This paper reviews the latest research progress of nanofluid flooding technology in the field of EOR. It systematically summarizes the oil displacement mechanisms of various nanomaterial-based oil displacement agents in improving recovery rates. It focuses on elaborating the application potential of SiO_2_, Al_2_O_3_, Fe_3_O_4_, carbon-based nanomaterials, and bio-based nano-flooding materials in enhancing oil recovery, particularly highlighting the broad prospects of bio-based nano-flooding materials in green and low-carbon oil extraction. Moreover, the field test results conducted in China, Colombia, Saudi Arabia, and Japan clearly demonstrate that this technology significantly improves oil recovery rates, which fully justifies further investment and promotion of this technology. However, there are still some key challenges, such as the unclear migration and retention of nanoparticles in the reservoir, the difficulty in controlling particle agglomeration and sedimentation, the incomplete understanding of adsorption mechanisms, and the limitations of nanoparticle numerical simulation techniques. Some of these require further economic assessment and justification. Future research should first consider optimizing the stability of nanofluids, developing new types of economic and environmentally friendly nanomaterials, and expanding the scale of field applications, ultimately clarifying the long-term potential of nanofluid flooding as a feasible, sustainable, and commercially competitive method for EOR.

## Figures and Tables

**Table 1:** Summary of previous studies on EOR by nanofluid in the past decade.

Base Fluid	Nanoparticle	IFT	Wettability	Rock	EOR (%)	Ref.
NaCl solution	Al_2_O_3_ and SiO_2_ nanoparticles	Basically consistent with brine	Al_2_O_3_: 121.3–76.5° SiO_2_: 135.8–85.6°	Berea sandstone Porosity: 21.6% Permeability: 135 mD	NA	[35]
Low-salinity water	Mesoporous SiO_2_	25.2–8.064 mN/m	127.4–20.7°	Montmorillonite	22–23%	[36]
NaCl solution	SiO_2_ + Graphene oxide nanoparticles	34.5–25.5 mN/m	159.6–66.4°	Carbonate rocks	15.50%	[37]
NaCl solution	ZrO_2_ + NiO nanoparticles	NA	88–48°	Carbonate rocks	NA	[38]
NaCl solution	SiO_2_ nanoparticles	NA	54–22°	Sandstone Porosity: 13–15% Permeability: 5–20 mD	10%	[39]
NaCl solution	TiO_2_, MgO, and Al_2_O_3_ nanoparticles	NA	TiO_2_: 136.73–37.18° MgO: 136.73–56.79° Al_2_O_3_: 136.73–49.21°	Carbonate rocks	TiO_2_: 30% MgO: 18% Al_2_O_3_: 25%	[40]
HCl and NaCl solution	Modified SiO_2_ nanoparticles	28–27.8 mN/m	NA	Carbonate rocks	40%	[41]
NaOH solution	SiO_2_ nanoparticles	NA	75–27°	Quartz	NA	[42]

NA: Not available, indicating no data or not determined.

**Table 4:** Summary of previous studies on EOR using bio-based nano oil displacement agents [13,15,21,43,78].

Material Type	Main Components	Main Performance Characteristics	EOR
Hybrid biopolymer-nanoparticle systems	Natural polymers (such as guar gum, xanthan gum) + nanoparticles (SiO_2_, Al_2_O_3_, TiO_2_)	Improve viscosity retention, enhance temperature and salt resistance, change rock wettability, and reduce oil–water interfacial tension	Adding 0.1 wt% SiO_2_ to the wt% gum arabic solution increased the recovery rate by an additional 14.4%
Microbial synthesis of nanocomposites	Synthesis of extracellular polysaccharides (metabolic products of Bacillus subtilis) and silver nanoparticles (E-Ag NPs)	The nanoparticles have high dispersion stability (with a Zeta potential reaching −43.4 mV) and can effectively reduce the interfacial tension and alter the wettability.	The spontaneous imbibition recovery rate reached 54.32%, and the core displacement recovery rate increased by 16.33%.
Biomass-based carbon nanomaterials	Surface-modified nanocellulose (12–25 wt%)	High specific surface area, adjustable surface chemistry, improved wettability, and stable viscosity.	Recovery rate of graphene-polymer nanocomposite materials is approximately 10% higher than that of pure polymer flooding
Biologically modified carbon nanomaterials	Biomass-derived carbon dots, carbon nanotubes, graphene. and their derivatives	Outstanding temperature resistance (150 °C), salt resistance (mineralization degree 118,913 mg/L), and a unique “simultaneous displacement” mechanism.	The fluid flow diversion rate in the low-permeability zone has increased by 10%
Plant-derived nanocomposites	Combination of plant extracts (such as eucalyptus and walnut shells) with nanofluids (such as xanthan gum/magnetite/SiO_2_)	Effectively reduce interfacial tension and contact angle	Recovery rate can reach 60–64%, superior to the 46% of the basic nanofluid.

**Table 5:** Application of nanofluid oil displacement agents in reservoirs with different characteristics [50,141,142,143].

Reservoir Type	Technical Adaptability	EOR Mechanisms	Application
Conventional sandstone	Medium and high water-cut oil reservoirs	Change the wettability of rocks, reduce the interfacial tension between oil and water, and strip the oil film in the pores.	Well Pu63-Slant672 of the Seventh Oil Production Plant of Daqing Oilfield (Nanometer Scissors Technology)
Low permeability	Inability to inject; inability to drive in reservoirs	Nanometer-scale advantages, penetrating micro-pore throats and being lipophilic and hydrophobic	The ultra-low permeability oil reservoir in the Bai Ma Central Area of Changqing Oilfield; “Nanometer Water Flooding” of China National Petroleum Corporation (Xinjiang, Changqing, etc.)
Carbonate rock	Fractured type best, followed by fracture-cavity type, and limited increase.	Nanoemulsion: reducing interfacial tension, emulsification; nanospheres: temporarily plugging high-permeability channels, fluid flow diversion	The fracture-cavity reservoirs in Tahe Oilfield (Nanometer Black Card Technology)
Heavy oil reservoir	Significantly reduce viscosity, improve fluidity, and achieve “cold production”	Emulsification for viscosity reduction (viscosity reduction rate up to 90%); removal of asphaltene and resin deposits	NA
Fractured reservoir	Coordinated regulation of fractures and matrix	Fracture flow regulation and matrix enhanced injection	Oil reservoirs in the Ordos Basin
Cavity-type reservoir	Applicable and with good well group connectivity	Emulsification and wetting reversal; complex oil reservoirs	The weathering crust-dark river composite well group in Tahe Oilfield

NA: Not available, indicating no data or not determined.

**Table 6:** The technical research progress of novel nano-oil displacement materials in the past five years.

Research Direction	Main Research Achievements	Key Technical Indicators	Advantages Compared with Traditional Technologies	Ref.
Janus-structured nanomaterials	Synthesis and oil displacement application of Janus SiO_2_ nanoparticles	Interfacial tension reduced to 0.067 mN/m; oil recovery increased by 25.41%	Significantly higher oil displacement efficiency than unmodified nanoparticles; excellent dispersion stability	[61]
Two-dimensional nanosheet materials	Field test of ODA-MoS_2_ nanosheet nanofluid	Concentration of 0.005 wt%; crude oil production increased; water cut decreased	High oil displacement efficiency achieved at low concentration, reducing costs	[166]
Bio-based nanocomposites	Microbial coupling silica nanocomposites	Zeta potential of −43.4 mV; spontaneous imbibition oil recovery of 54.32%	Green and biodegradable; excellent dispersion stability	[167]
Deepening of oil-displacement mechanism	Quantitative study on structural disjoining pressure (SDP) of nanosheets	SDP of 50 kPa generated by 0.005 wt% nanosheets	Solves the problem of high concentration requirement for traditional nanoparticles	[168]
Industrial application	Field test of modified SiO_2_ composite system	Oil recovery increased by 16.33%; cost reduced by 20%	Significantly improved economic feasibility; suitable for low-permeability reservoirs	[169]
Cost optimization	Preparation of nanocomposites from fly ash	Production cost reduced by 40%	Utilizes industrial by-products; green, environmentally friendly, and low-cost	[170]

## Data Availability

No new data were created or analyzed in this study. Data sharing is not applicable to this article.
